# Kai-Xin-San series formulae alleviate depressive-like behaviors on chronic mild stressed mice via regulating neurotrophic factor system on hippocampus

**DOI:** 10.1038/s41598-017-01561-2

**Published:** 2017-05-03

**Authors:** Yue Zhu, Cheng Chao, Xiuzhu Duan, Xiaoxuan Cheng, Pei Liu, Shulan Su, Jinao Duan, Tina Tingxia Dong, Karl Wah-Keung Tsim

**Affiliations:** 1 Jiangsu Key Laboratory for High Technology Research of TCM Formulae and Jiangsu Collaborative Innovation Center of Chinese Medicinal Resources Industrialization, State Key Laboratory Cultivation Base for TCM Quality and Efficacy, Nanjing University of Chinese Medicine Nanjing University of Chinese Medicine, Nan Jing, Jiangsu Province China; 2 Division of Life Science and Center for Chinese Medicine, Division of Life Science, The Hong Kong University of Science and Technology, Clear Water Bay, Hong Kong China

## Abstract

Kai-xin-san (KXS) is a famous Chinese medicinal formula applied for treating stress-related psychiatric diseases with the symptoms such as depression, forgetfulness and dizziness. In clinic, the composition ratio of KXS is always varied and KXS series formulae are created. Here, we aim to compare the anti-depressive effect of different ratios of KXS and reveal its action mechanism on regulation of neurotrophic factor system. Firstly, daily intra-gastric administration of chemically standardized extracts of KXS series formulae for seven days significantly alleviated the depressive symptoms of chronic unpredictable mild stressed mice displayed by enhanced sucrose consumptions and decreased immobile time of forced swimming coupled with increased locomotor activities. KXS might fulfill this effect by up-regulating the expressions of NGF, BDNF and Trk receptors in hippocampus, which were confirmed by the treatment of corresponding blockers tPA-stop and K252a. The ratio with higher amounts of Ginseng Radix et Rhizoma and Polygalae Radix exerted most profound effect on anti-depression and regulation enzymes in metabolic pathway of neurotrophic factors. These findings suggested that KXS was beneficial for enhancing supplies, up-regulating receptors, and restoring the dysfunction of metabolic pathway of neurotrophic factors, which might account for its anti-depression effect.

## Introduction

With the growing incidence in the recent years, major depression disorder has become one of the most common psychiatric disorders. WHO also reports that depression disorder may rank as the second leading cause of world disabilities by 2020 and becomes the largest global burden of disease by 2030^1^. The depressed patients always experience pleasure loss, fatigue, disturbance of sleep and appetite, difficulty in decisions, and prone to commit suicide, which bring great discomfort not only to the patients themselves but also to their families, friends and the whole society.

Within etiological hypotheses of depression, dysfunction of neurotrophic factor regulation is an important one^2^. Neurotrophic factors, in short neurotrophins, are a family of proteins including nerve growth factor (NGF), brain-derived neurotrophic factor (BDNF), neurotrophin-3 (NT-3), neurotrophin-4/5 (NT-4/5) and so on. Neurotrophins are responsible for the growth and survival of neurons during development and for maintenance of adult neurons. They exert these functions by interacting with two entirely distinct classes of receptors, Trks and p75^NTR^. NGF binds to TrkA, BDNF and NT-4/5 bind to TrkB and NT-3 binds to TrkC with high affinity. NT-3 also interacts with TrkA and TrkB, albeit with less efficiency. For p75^NTR^, pro-neurotrophins bind to this receptor to elicit neuronal apoptosis, contrary to the nourish function elicited by Trk receptors^3^. Among neurotrophins, BDNF has been regarded as an important common mediator of depression. Reduction of BDNF has been found both in amygdala and hippocampus and serum of patients with major depression^4^. In therapies, BDNF transcription can be activated by anti-depressants and the depressive state is relieved^5^. In animal studies, BDNF reduction is also found in hippocampus and closely related to depressive-like behaviors^6, 7^. In addition to BDNF, NGF expression is also found to be decreased in hippocampus, prefrontal cortex and amygdala by stress and can be reversed by anti-depressants^8, 9^. Since neurotrophins are proteins difficult to penetrate blood brain barrier and the direct infusion of NGF and BDNF into hippocampus is unrealistic in clinic, induction of endogenous neurotrophin expressions is critical for development of anti-depressants. Some monoamine reuptake inhibitors applied in clinic, like fluoxetine, escitalopram and venlafaxine, have be found to increase BDNF expression on depressive-like rodent models^10–12^. However, the non-ignorable reality is that over 40% of patients has no response to these popular anti-depressants. More astonishing, fluoxetine has been found to increase the odds of suicide by 50% in children and adolescents, not to mention other side effects^13^. In this situation, traditional Chinese medicine formulae, a complex system with multiple compounds and action targets, have been paid more attention in development of anti-depressants.

Kai-Xin-San (KXS), firstly described in *Beiji Qianjin Yaofang* < *Thousand Formulae for Emergency* > by Sun Si-miao of Tang Dynasty in 652 A.D, is a famous TCM formula applied for treating mental disorders. KXS is comprised of four herbs: Ginseng Radix et Rhizome (root and rhizome of *Panax ginseng* C. A. Mey., Renshen, GR), Polygalae Radix (root of *Polygala tenuifolia* Wild., Yuanzhi, PR), Acori Tatarinowii Rhizoma (rhizome of *Acorus tatarinowii* Schott, Shichangpu, ATR), and Poria (sclerotium of *Poria cocos* (Schw.) Wolf, Fuling, PO) and the ratio is 1:1:25:50 (RG: RP: RAT: PO, K-652). In TCM clinic, the formula is frequently to be re-arranged by combinations or dosages according to the results from syndrome differentiation and series formulae are created. Therefore, other two ratios of KXS have widely been applied in clinic, one named Ding-Zhi-Wan (DZW-652, D-652) also described in *Beiji Qianjin Yaofang* with ratio of 3:2:2:3 and another named KXS-984 (K-984), recorded in *Yi Xin Fang* compiled by Nima Yasunori from Japan in 984 A.D. with ratio of 1:1:1:2. No matter the ratio varied, KXS is prescribed to treat a mental disorder with these symptoms: unhappiness, morbid forgetfulness and dizziness, which is very similar to major depression disorder. Until now, KXS is still applied for the treatment of depression in clinic and has been reported to relieve the depressive symptoms of major depression disorder patients, as also revealed in animal studies^14, 15^. For exploration of the action mechanism of KXS, most of the researches focus on neurotransmitter regulation^16, 17^ while the effect on neurotrophin regulation is seldom explored^18^.

In our previous studies, we found that one ratio of KXS series formulae, named K-652 (1:1:25:50), could significantly alleviate the depressive like symptoms on chronic unpredictable mild stress (CUMS) induced depressive-like rats by restoring deficiency of monoamine neurotransmitters and increasing mRNA expressions of neurotrophins and tyrosine kinase (TrK) receptors^14^. Therefore, we hope to further clarify the relationship between anti-depressive effect and neurotrophin system regulation of KXS in current studies. Firstly, KXS series formulae were applied on a CUMS-induced depressive-like mice model and the effect of KXS on expressions of neurotrophins and Trk receptors in hippocampus were evaluated. Afterwards, the mRNA expressions of proteins accounting for metabolic pathway of neurotrophic factors would be evaluated and the corresponding inhibitors were applied to further clarify the mechanism.

## Results

### KXS series formulae alleviate the depressive-like symptoms of chronic unpredictable mild stressed mice

**Figure 1:**
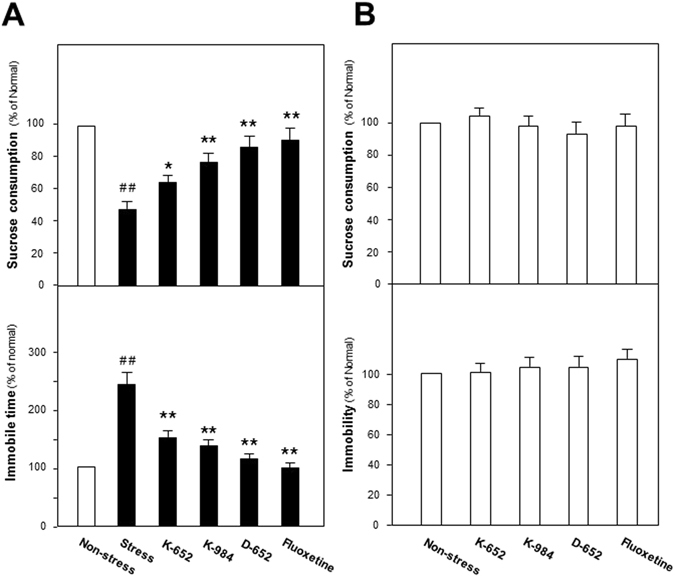
Effect of KXS series formulae treatment on the behaviors of CUMS-treated mice. (**A**) After four weeks of CUMS treatment, all groups of mice were employed for behavior evaluation tests, including the sucrose preference test, forced swimming test. Fluoxetine at daily dosage of 4 mg/kg was set as the positive control. KXS series treatment was set for three groups, K-652, K-984, D-652, all at 10 g/kg. (**B**) Non-stressed mice were treated with fluoxetine and KXS. The period and dosage of treatment were same to that of CUMS treated groups. Afterwards, same behavior evaluation tests were applied. Values are expressed in the percentage of non-stressed group as Mean ± SEM (n = 8). Comparisons between groups were carried out by a one-way ANOVA followed by a post-hoc Bonferroni test. ^#^p < 0.05, ^##^p < 0.01 (compared with non-stressed group); *p < 0.05, **p < 0.01 (compared with stressed group).

Details of CUMS procedures were described in Material and method section and displayed in Supplementary Fig. 2. After 4 week CUMS cycles finished, all of mice were given sucrose consumption test and forced swimming test, which mimicked symptoms of anhedonia (loss of pleasure) and despair (loss of hope) of depression patients. In Fig. 1A, sucrose consumption of CUMS group animals was significantly reduced after CUMS exposures (47% of non-stressed control group, compared with non-stressed control group, F_(5,47)_ = 23.34, p < 0.01) while significantly increased by fluoxetine treatment (87% of non-stressed control group, compared with stressed group, F_(5,47)_ = 23.34, p < 0.01). Similarly, the increased immobile time in forced swimming tests due to CUMS exposure (242% of non-stressed control group, compared with non-stressed control group, F_(5,47)_ = 57.02, p < 0.01) was reversed to be close to normal level by fluoxetine (99% of non-stressed control group, compared with stressed control group, F_(5,47)_ = 57.02, p < 0.01). In KXS series formulae treated CUMS mice, as shown in Fig. 1A, sucrose consumption was significantly increased compared with CUMS stressed group (66% of non-stressed control group for K-652, p < 0.05; 76% of non-stressed control group for K-984, p < 0.01; 83% of non-stressed control group or D-652, p < 0.01). On forced swimming test, KXS series formulae significantly shortened the immobile time compared with CUMS stressed group (153% of non-stressed control group for K-652, 137% of non-stressed control group for K-984, 122% of non-stressed control group for D-652, F_(5,47)_ = 57.02, p < 0.01). Meanwhile, shown in Fig. 1B, the treatment of KXS or fluoxetine in normal mice, did not show any significant effect on the sucrose consumption (F_(4,39)_ = 1.61, p = 0.195) and swimming immobile time (F_(4,39)_ = 1.27 p = 0.299). Body weights of mice were also measured and CUMS procedures significantly reduced the body weight of mice compared with non-stressed group. However, the treatment of KXS and fluoxetine afterwards did not alter this tendency (Data were not shown).

**Figure 2:**
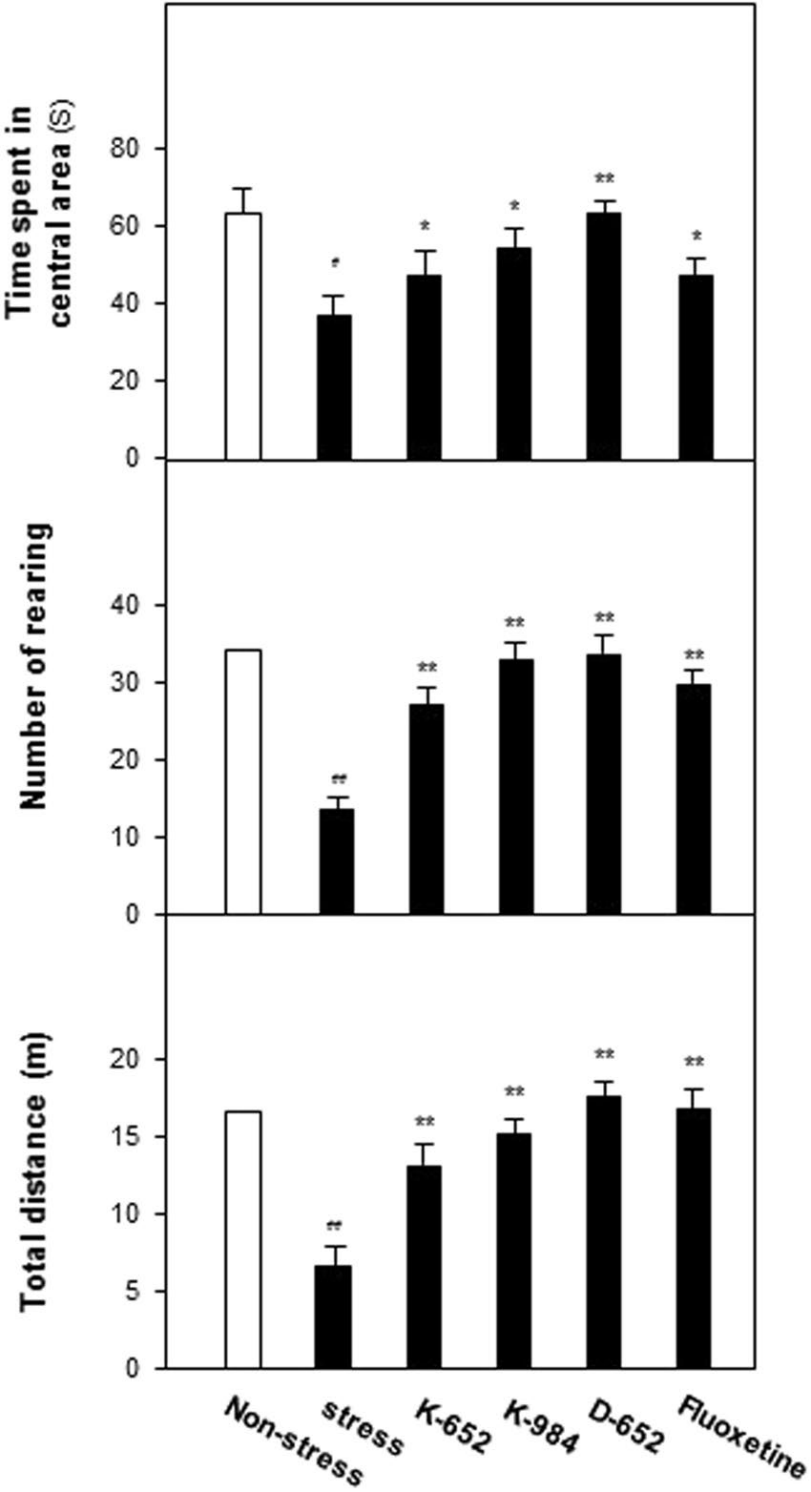
KXS series formulae exerted antidepressant actions in open field tests of CUMS-treated mice. After four weeks of CUMS exposure and one week of KXS series formulae treatment, all groups of mice were employed for open field tests, including time spent in central area, rearing number and total travel distance. Fluoxetine at daily dosage of 4 mg/kg was set as the positive control. KXS series formulae treatment was set for three groups, K-652, K-984, D-652, all at 10 g/kg. Values are expressed in the percentage of non-stressed group as Mean ± SEM (n = 8). Comparisons between groups were carried out by a one-way ANOVA followed by a post-hoc Bonferroni test. ^#^p < 0.05, ^##^p < 0.01 (compared with non-stressed group); *p < 0.05, **p < 0.01 (compared with stressed group).

Besides, open field test was also applied to evaluate the effect of KXS series formulae on locomotor activities of mice. From Fig. 2, CUMS exposures significantly decreased the time spent in central area, rearing numbers and total distances compared with non-stressed control group. Treatment of KXS series formulae significantly enhanced the time spent in central area (F_(5,47)_ = 14.58, p < 0.01), rearing numbers (F_(5,47)_ = 16.51, p < 0.01) and total distances (F_(5,47)_ = 11.17, p < 0.01) compared with CUMS group. Taken these data together, the depressive-like behaviors like anhedonia, despair and decreased locomotor activities induced by CUMS exposures could be ameliorated by treatment of KXS series formulae. Interestingly, the alleviated tendency of depressive-like symptoms was in accordance with the increased amounts of GR and PR in KXS series formulae and D-652, with the highest ratio of GR and PR, exerted the best efficacy.

### KXS series formulae restore the decreased levels of neurotrophic factors in hippocampus of CUMS-induced depressive mice

**Figure 3:**
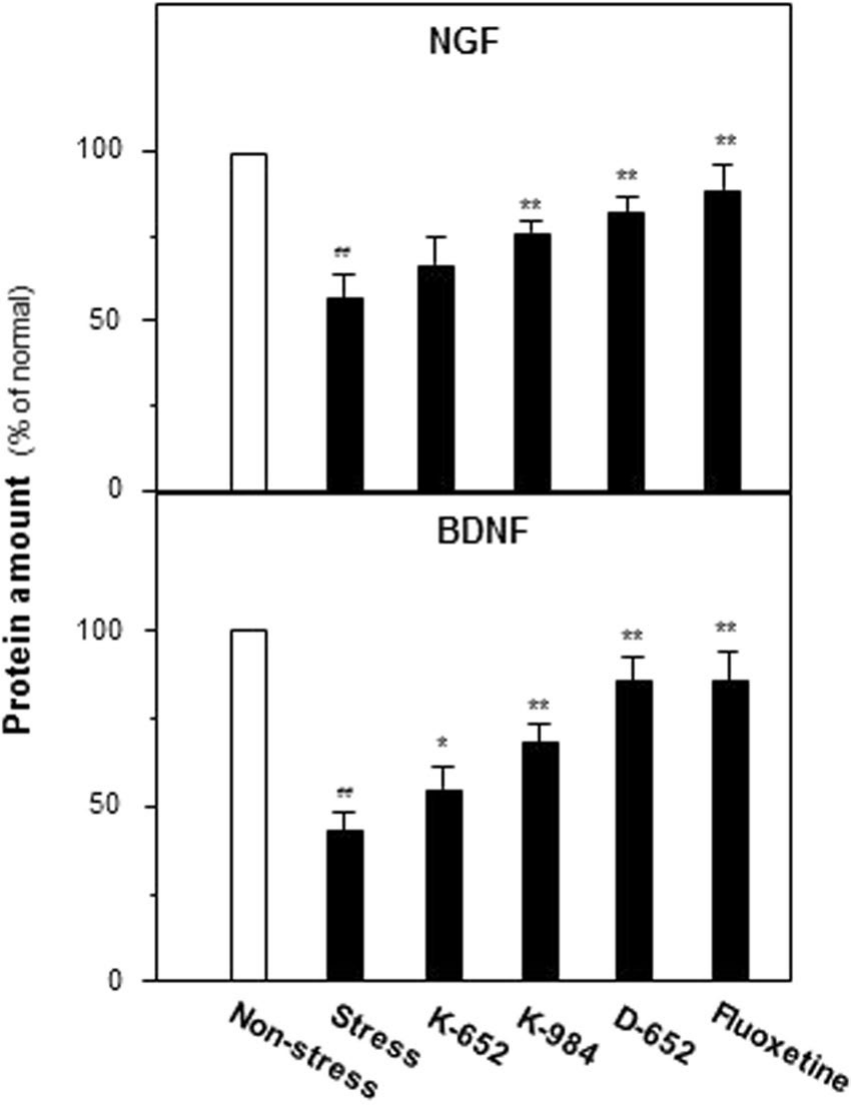
Effect of KXS series formulae treatment on expressions of neurotrophic factors in hippocampus of CUMS-treated mice by ELISA analysis. The treatment of KXS series formulae and fluoxetine in the mice were same as that in Fig. 1. The hippocampus tissues were collected after four-week treatment and the amounts of NGF were analyzed by ELISA kits. Comparisons between groups were carried out by a one-way ANOVA followed by a post-hoc Bonferroni test. Values are expressed in the percentage of non-stressed group as Mean ± SEM (n = 8). ^#^p < 0.05, ^##^p < 0.01 (compared with non-stressed group); *p < 0.05, **p < 0.01 (compared with stressed group).

After behavioral evaluation, the amounts of NGF and BDNF were determined in hippocampus of different groups of mice by ELISA method. In Fig. 3, CUMS exposure significantly decreased expressions of NGF (56% of non-stressed control group, compared with non-stressed control group, F_(5,47)_ = 34.85, p < 0.01) and BDNF (48% of non-stressed control group, compared with non-stressed control group, F_(5,47)_ = 215.49, p < 0.01) in hippocampus. Fluoxetine increased expressions of NGF (88% of non-stressed control group, compared with stressed group, F_(5,47)_ = 34.85, p < 0.01) and BDNF (82% of non-stressed control group, compared with stressed group, F_(5,47)_ = 215.49, p < 0.01). For KXS formulae, K-984 and D-652 significantly up-regulated protein levels of NGF (76% of non-stressed control group for K-984, 83% of non-stressed control group for D-652, both compared with stressed group, F_(5,47)_ = 34.85, p < 0.01) and BDNF (70% of non-stressed control group for K-984 and 87% of non-stressed control group for D-652, both compared with stressed group, F_(5,47)_ = 215.49, p < 0.01). For K-652, it did not up-regulate expression of NGF (52% of non-stressed control group, compared with stressed group, p = 0.41) and BDNF (52% of non-stressed control group, compared with stressed group, p = 0.45).

**Figure 4:**
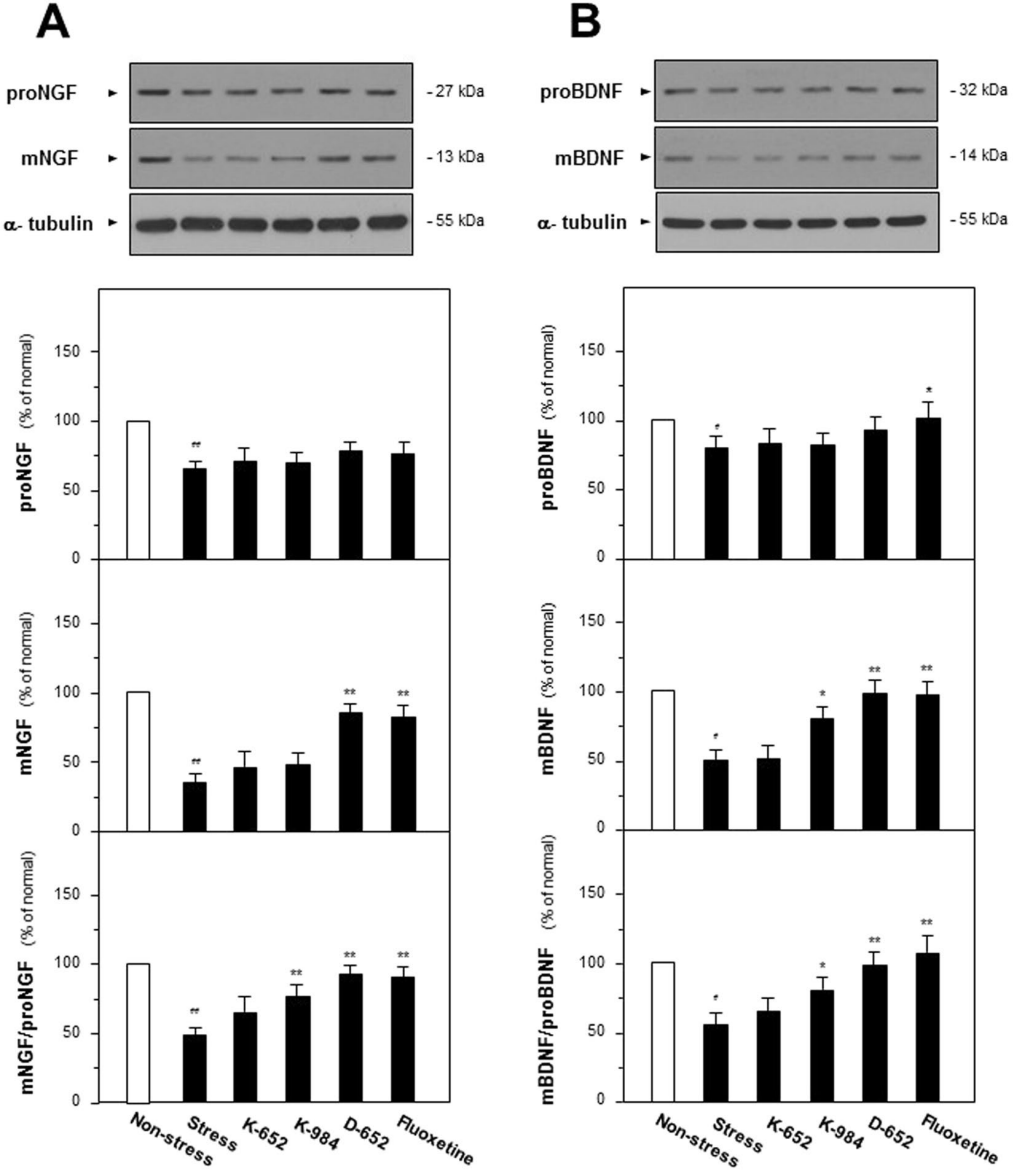
Effect of KXS series formulae treatment on the protein expressions of neurotrophic factors and its precursors in hippocampus of CUMS-treated mice by western analysis. (**A**) The treatment of KXS series formulae and fluoxetine in the mice were same as that in Fig. 1. The hippocampus tissues were collected after four-week treatment and the expressions of proNGF and NGF were analyzed by western blot analysis. α-tubulin was used as the loading control. Afterwards, the ratio of mNGF to proNGF was calculated. (**B**) Expressions of proBDNF and BDNF were determined and the ratio of BDNF to proBDNF was calculated as same as that of NGF. Comparisons between groups were carried out by a one-way ANOVA followed by a post-hoc Bonferroni test. Values are expressed in the percentage of non-stressed group as Mean ± SEM (n = 8). ^#^p < 0.05, ^##^p < 0.01 (compared with non-stressed group); *p < 0.05, **p < 0.01 (compared with stressed group).

Since the mature forms and precursors were unable to be distinguished by ELISA analysis, the expressions of precursors of neurotrophins: proNGF and proBDNF were determined by western blot analysis, and then the ratios of its mature forms mNGF and mBDNF to precursors were calculated. As shown in Fig. 4, the expressions of proNGF and proBDNF were down-regulated in CUMS group (67% for proNGF, compared with non-stressed group, F_(5,47)_ = 6.97, p < 0.01 and 79% for proBDNF of non-stressed group, compared with non-stressed group, F_(5,47)_ = 3.83, p < 0.01) and all of the drug treatment groups did not exerted the up-regulated tendencies. On the contrary, the expressions of mNGF were down-regulated in CUMS group (36% of non-stressed group, compared with non-stressed group, F_(5,47)_ = 43.13, p < 0.01) while they were up-regulated after treatment of fluoxetine (83% of non-stressed group, compared with stressed group, F_(5,47)_ = 43.13, p < 0.01) and D-652 (88% of non-stressed group, compared with stressed group, F_(5,47)_ = 43.13, p < 0.01), which was in line with the results of ELISA analysis. By calculating the ratio of mNGF to proNGF, CUMS procedures inhibited the transformation of proNGF to mNGF (48% of non-stressed group, compared with non-stressed group, F_(5,47)_ = 20.60, p < 0.01) and this tendency could be reversed by the treatment of KXS, especially D-652 (91% of non-stressed group, compared with stressed group, F_(5,47)_ = 20.60, p < 0.01). K-652 only showed the similar up-regulation tendency to K-984 and D-652, however no significant difference was found (65% of non-stressed group, compared with stressed group, F_(5,47)_ = 20.60, p = 0.61). The similar phenomena could also be found in calculation of mBDNF to proBDNF. Here, KXS formulae with higher amount of GR and PR, such as K-984 and D-652, had better effect in increasing expressions of NGF and BDNF in hippocampus via enabling transformation of precursor to mature form.

### KXS series formulae regulate synthesis and degradation of neurotrophic factors

Based on the phenomena of KXS modulating the processing of neurotrophins, the mRNA levels of proteins relating to processing were determined by quantitative PCR analysis. The critical enzymes can be categorized into two groups: plasminogen, tissue plasminogen activator (tPA) and neuroserpin for synthesis while matrix metallopeptidase 9 (MMP-9) and tissue inhibitor of metalloproteinase 1 (TIMP-1) for degradation. These proteins also act on processing of BDNF^19^.

**Figure 5:**
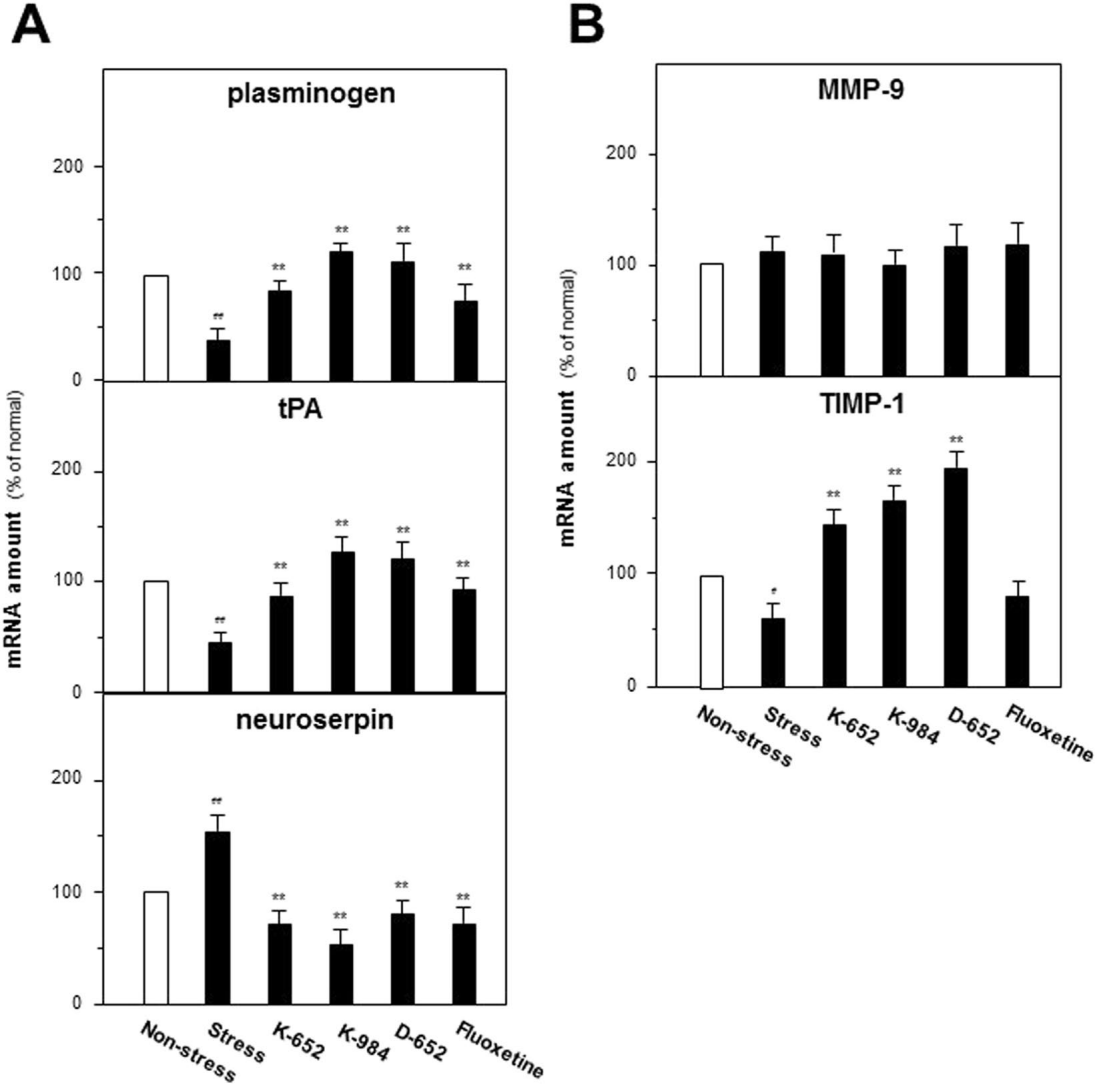
KXS series formulae regulate mRNA expressions of proteins relating to metabolic pathway of neurotrophic factors in hippocampus of CUMS-treated mice. (**A**) The treatment of KXS series formulae and fluoxetine in the mice were same as that in Fig. 1. The hippocampus tissues were collected after one-week treatment and mRNA expression levels of proteins relating to synthesis of neurotrophic factors were analyzed. (**B**) Drug treatment and sample preparations were same as (**A**) and mRNA expression levels of proteins relating to degradation of neurotrophic factors were analyzed. Comparisons between groups were carried out by a one-way ANOVA followed by a post-hoc Bonferroni test. Values are expressed in the percentage of non-stressed group as Mean ± SEM (n = 8). ^#^p < 0.05, ^##^p < 0.01 (compared with non-stressed group); *p < 0.05, **p < 0.01 (compared with stressed group).

The effects of KXS series formulae extracts on mRNA expressions of proteins accounting for synthesis and degradation of neurotrophins could be found in Fig. 5. For enzymes related to neurotrophin synthesis, CUMS exposures significantly decreased the mRNA levels of plasminogen (37% of non-stressed group, compared with non-stressed group, F_(5,47)_ = 43.72, p < 0.01) and tPA (41% of non-stressed group, compared with non-stressed group, F_(5,47)_ = 33.64, p < 0.01) while increased neuroserpin (152% of non-stressed group, compared with non-stressed group, F_(5,47)_ = 40.43, p < 0.01). KXS series formulae up-regulated expressions of plasminogen (87% for K-652, 110% for K-984 and 107% for D-652 of non-stressed group, compared with stressed group, F_(5,47)_ = 43.72, p < 0.01) and tPA (88% for K-652, 125% for K-984 and 122% for D-652 of non-stressed group, compared with stressed group, F_(5,47)_ = 33.64, p < 0.01) while down-regulated neuroserpin expression (74% for K-652, 51% for K-984 and 78% for D-652 of non-stressed group, compared with stressed group, F_(5,47)_ = 40.43, p < 0.01). Though not showing profound effects on protein levels of precursors and mature forms of neurotrophins, K-652 could also regulate mRNA expressions of these synthesis enzymes compared with D-652.

For degradation related enzymes, TIMP-1 mRNA levels were significantly down-regulated by the CUMS exposures in hippocampus (58% of non-stressed group, compared with non-stressed group, F_(5,47)_ = 34.32, p < 0.01) while up-regulated by KXS treatment (147% for K-652, 161% for K-984 and 198% for D-652 of non-stressed group, compared with stressed gro up, F_(5,47)_ = 34.32, p < 0.01). For MMP9, no significant statistical difference could be found between groups (F_(5,47)_ = 1.83, p = 0.128).

**Figure 6:**
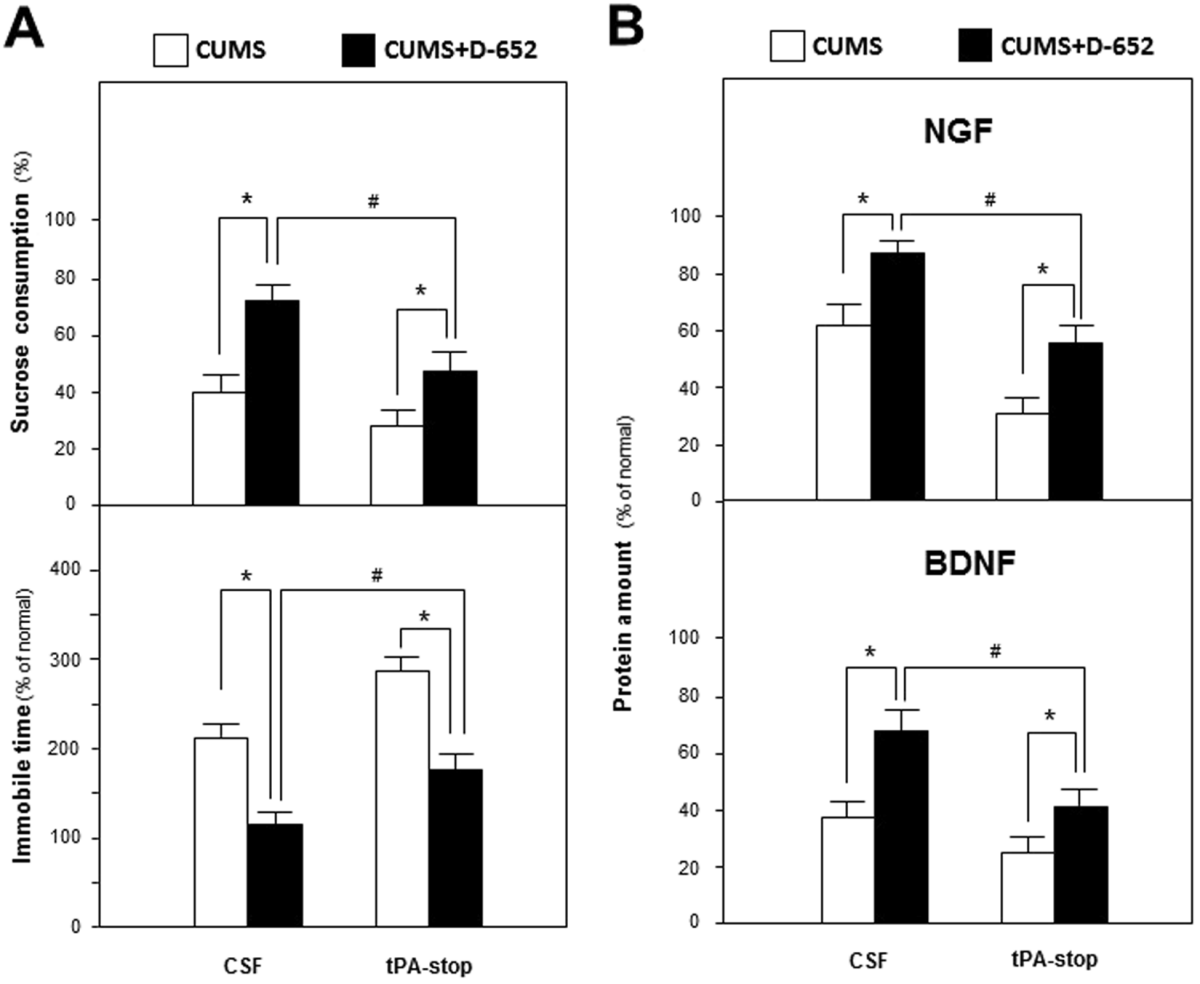
The blocker of tPA attenuates anti-depressive actions of KXS in the behavioral tests and expressions of neurotrophic factors of hippocampus of CUMS-treated mice. (**A**) At the end of CUMS exposures, one group of the mice were treated with D-652 and tPA-stop simultaneously. D-652 was orally treated for 7 days and tPA-stop (dissolved in artificial CSF) was transcranial injected into hippocampus for 3 days. Then, sucrose preference test and forced swimming test were carried out. The CUMS mice only injected with artificial CSF and treated with combination of CSF and D-652 were applied for contrast. (**B**) Amounts of NGF and BDNF were determined in hippocampus of mice as treated in (**A**). Comparisons between groups were carried out by a two-way ANOVA followed by a post-hoc Bonferroni test. Values are expressed in the percentage of non-stressed group as Mean ± SEM (n = 8). ^#^p < 0.05, ^##^p < 0.01 (comparison between group treated with CUMS/CSF plus D-652 and group treated with CUMS/CSF plus D-652 and tPA-stop); *p < 0.05, **p < 0.01 (comparison between group treated with CUMS/CSF and group treated with CUMS/CSF plus D-652; comparison between group treated with CUMS/CSF plus tPA-stop and group treated with CUMS/CSF plus D-652 and tPA-stop).

Based on mRNA expression results, one synthetic blocker of tPA named tPA-stop, was applied to determine whether KXS series formulae regulated neurotrophin synthesis via tPA. D-652, exerting most profound anti-depressive effect and inducing expressions of NGF and BDNF in hippocampus, was selected among three KXS formulae. From Fig. 6A, the ameliorated depressive symptoms in D-652 treatment group, in presence of increased sucrose consumption (72% of non-stressed group, compared with CSF injected stressed group, p < 0.05) and decreased immobile swimming time (110% of non-stressed group, compared with CSF injected stressed group, p < 0.05) was attenuated by the simultaneous tPA-stop treatment. On sucrose consumption test, a two-way ANOVA revealed significant differences for the tPA stop-treatment (F_(3,31)_ = 75.17, p < 0.01), D-652 treatment (F_(3,31)_ = 166.32, p < 0.01) and tPA stop × D-652 interaction (F_(3,31)_ = 21.89, p < 0.01). The treatment of tPA-stop alone did not affect the sucrose consumption in non-D-652 treated CUMS/CSF group mice (compared with CUMS/CSF group, p = 0.05), but significantly decreased it in the D-652 treated CUMS/CSF group mice (compared with D-652 treated CUMS/CSF group, p < 0.01). Meanwhile, simultaneous treatment of D-652 and tPA-stop in CUMS/CSF group mice also increased sucrose consumption compared with single tPA-stop treated CUMS/CSF group mice (p < 0.05). On forced swimming test, a two-way ANOVA revealed significant differences for the tPA stop-treatment (F_(3,31)_ = 75.76, p < 0.01) and D-652 treatment (F_(3,31)_ = 183.51, p < 0.01) but not tPA stop × D-652 interaction (F_(3,31)_ = 0.071, p = 0.792). The treatment of tPA stop significantly increased the immobile swimming time in non-D-652 treated CUMS/CSF group mice (compared with CUMS/CSF group, p < 0.01) and decreased that in D-652 treated CUMS/CSF mice (compared with CUMS/CSF group, p < 0.05). Meanwhile, simultaneous treatment of D-652 and tPA-stop in CUMS/CSF group mice also decreased immobile swimming time compared with single tPA-stop treated CUMS/CSF group mice (p < 0.05).

In parallel, tPA-stop also attenuated the increased tendencies of NGF and BDNF by the treatment of D-652 on CUMS mice. Displayed in Fig. 6B, a two-way ANOVA revealed differences of NGF expression for the tPA stop-treatment (F_(3,31)_ = 58.24, p < 0.01), D-652 treatment (F_(3,31)_ = 40.19, p < 0.01) and tPA stop × D-652 interaction (F_(3,31)_ = 1.38, p = 0.25). The treatment of tPA stop significantly decreased NGF expressions in non-D-652 treated CUMS/CSF group mice (compared with CUMS/CSF group, p < 0.01) and increased that in D-652 treated CUMS/CSF mice (compared with D-652 treated CUMS/CSF group, p < 0.01). Meanwhile, simultaneous treatment of D-652 and tPA-stop also increased NGF expressions compared with single tPA-stop treated CUMS/CSF group mice (p < 0.05). Similar tendencies were also found for BDNF expressions. A two-way ANOVA revealed significant differences for the tPA stop-treatment (F_(3,31)_ = 113.62, p < 0.01) and D-652 treatment (F_(3,31)_ = 147.21, p < 0.01) and tPA stop × D-652 interaction (F_(3,31)_ = 19.08, p < 0.01). The treatment of tPA stop significantly decreased the BDNF expression in non-D-652 treated CUMS/CSF group mice (compared with CUMS/CSF group, p < 0.05) and increased that in D-652 treated CUMS/CSF group mice (compared with D-652 treated CUMS/CSF group, p < 0.05). Meanwhile, simultaneous treatment of D-652 and tPA-stop also increased BDNF expressions compared with single tPA-stop treated CUMS/CSF group mice (p < 0.05).

Based on these data, KXS might enhance the expressions of NGF and BDNF via regulating tPA system in stimulating synthesis of mNGF, which might account for its anti-depression effect.

### KXS series formulae up-regulate Trk receptors in hippocampus of CUMS-induced depressive mice

**Figure 7:**
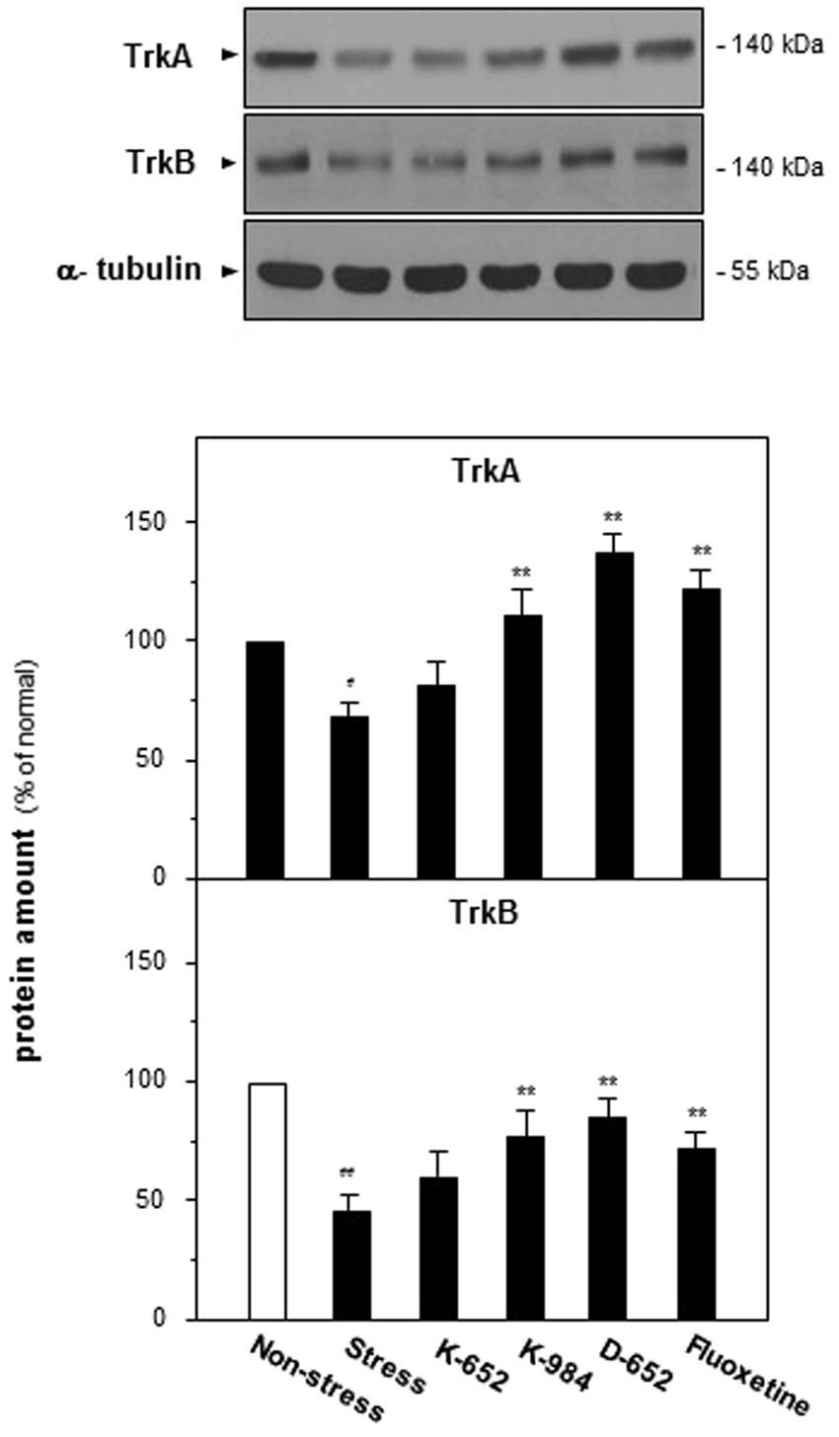
Effect of KXS series formulae treatment on the protein expressions of Trk receptors in hippocampus of CUMS-treated mice. The treatment of KXS series formulae and fluoxetine in the mice was same as that in Fig. 1. The hippocampus tissues were collected after one-week treatment and protein levels of TrkA and TrkB were analyzed. Comparisons between groups were carried out by a one-way ANOVA followed by a post-hoc Bonferroni test. Values are expressed in the percentage of non-stressed control group as Mean ± SEM (n = 8). ^#^p < 0.05, ^##^p < 0.01 (compared with non-stressed group), *p < 0.05, **p < 0.01 (compared with stressed group).

In addition to neurotrophins, TrkA and TrkB, the receptors of NGF and BDNF, were further explored on protein expressions via western blot analysis. From Fig. 7, decreased expressions of TrkA in CUMS group (68% of non-stressed group, compared with non-stressed group, F_(5,47)_ = 18.44, p < 0.01) were reversed by K-984 and D-652 (115% for K-984 and 126% for D-652 of non-stressed group, compared with stressed group, F_(5,47)_ = 18.44, p < 0.01). CUMS exposures also decreased TrkB expressions (48% of non-stressed group, compared with non-stressed group, F_(5,47)_ = 28.46, p < 0.01) while K-984 and D-652 increased TrkB expressions significantly (76% for K-984 and 81% for D-652 of non-stressed group, compared with stressed group, F_(5,47)_ = 28.46, p < 0.01).

**Figure 8:**
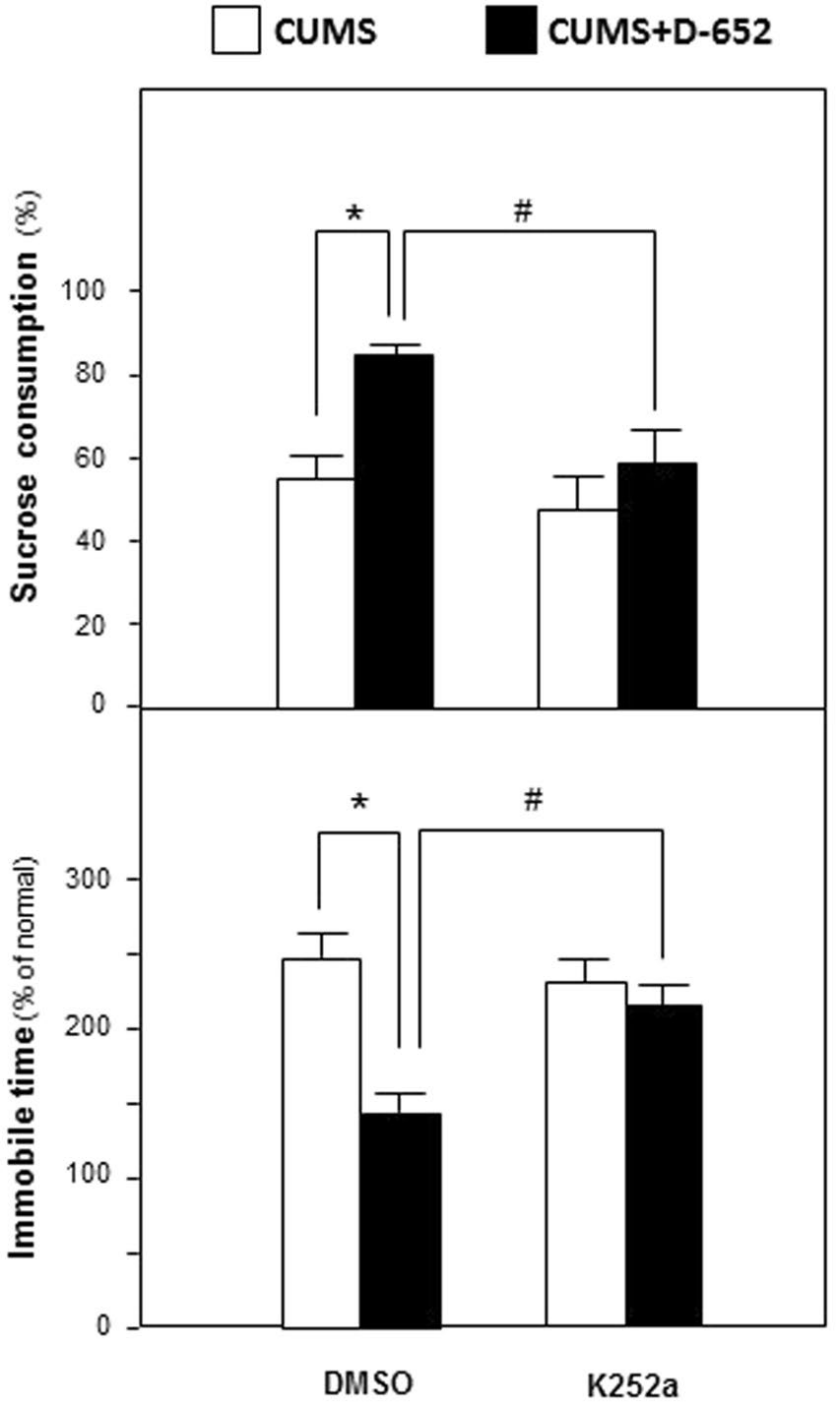
The blocker of Trk attenuates antidepressant actions of KXS in the behavioral tests of CUMS-treated mice. At the end of CUMS exposures, one group of the mice were treated simultaneously with D-652 and K252a for 7 days. D-652 was orally treated and K252a (dissolved in DMSO) was administered intraperitoneally. Then, sucrose preference test and forced swimming test were carried out. The CUMS mice only injected with DMSO and treated with combination of DMSO and D-652 were applied for contrast. Comparisons between groups were carried out by a two-way ANOVA followed by a post-hoc Bonferroni test. Values are expressed in the percentage of non-stressed control group as Mean ± SEM (n = 8). ^#^p < 0.05, ^##^p < 0.01 (comparison between group treated with CUMS/DMSO plus D-652 and group treated with CUMS/DMSO plus D-652 and K252a). *p < 0.05, **p < 0.01 (comparison between group treated with CUMS/DMSO and group treated with CUMS/DMSO plus D-652).

Afterwards, K252a, the antagonist of Trk receptors, was applied to evaluate whether KXS exerted anti-depression effect by regulating neurotrophin-Trk signaling pathway. From Fig. 8, D-652 increased sucrose consumption and decreased immobile time of mice in forced swimming test compared with that of CUMS group and these tendencies was attenuated by the treatment of K252a. A two-way ANOVA revealed significant differences for the K252a treatment (F_(3,31)_ = 54.12, p < 0.01), D-652 treatment (F_(3,31)_ = 66.70, p < 0.01) and K252a × D-652 interaction (F_(3,31)_ = 16.92, p < 0.01). The treatment of K252a alone did not affect the sucrose consumption in DMSO treated CUMS mice (compared with DMSO treated CUMS group, p = 0.18), but significantly decreased it in the D-652 treated CUMS/DMSO group mice (compared with DMSO treated CUMS group, p < 0.05). On forced swimming test, a two-way ANOVA revealed significant differences for the K252a-treatment (F_(3,31)_ = 27.16, p < 0.01), D-652 treatment (F_(3,31)_ = 87.12, p < 0.01) and K252a × D-652 interaction (F_(3,31)_ = 18.66, p < 0.01). The treatment of K252a alone did not affect the immobile time in DMSO treated CUMS mice (compared with DMSO treated CUMS group, p = 0.98), but significantly decreased it in the D-652 treated CUMS/DMSO mice (compared with DMSO treated CUMS group, p < 0.05).

These phenomena showed that the effect of D-652 on ameliorating depression-like behaviors of CUMS mice was attenuated by blocking Trk receptors. Therefore, neurotrophin-Trk signaling pathway might be crucial for KXS formulae exerting anti-depression effect in CUMS induced depression-like mice.

## Discussion

In TCM anti-depression researches, KXS is among the most popular formulae. Previous studies confirm that KXS shows positive effect in enhancing memory or alleviating depression-like symptoms on animal models^20–25^. As to its action mechanism, most of research articles focus on regulation of monoamine neurotransmitter and stress hormone system^16, 17, 26, 27^, while only a few paper on neurotrophin system^18, 28^.

The published research articles on neurotrophin regulation of KXS mainly describe the phenomena of KXS inducing expressions of neurotrophins. In *in vivo* studies, one compatible ratio of KXS, i.e. K-652 in current research, induces mRNA expression levels of neurotrophins and Trk receptors in total brain of CUMS rats^14^. Another *in vitro* study reveals that KXS series formulae induce neurotrophin expressions in rat cortical astrocyte primary cultures^29^. However, the concrete regulation mechanism is never reported. In current studies, the specific targets for KXS affecting synthesis and degradation of neurotrophins were revealed and tPA might be an important one based on our findings. In central nervous system, dysregulation of tPA/plasminogen system is found to be important in pathology of depression and development of anti-depressants^30, 31^ because regulation of tPA/plasminogen system is closely related to neurotrophin processing. On a clinical study by detecting BDNF, proBDNF and receptors in the sera and lymphocytes of depression patients, the levels of proBDNF, sortilin and p75^NTR^ are higher while mature BDNF and TrkB levels are lower compared with that of healthy controls, which implies that the balance between the proBDNF/p75^NTR^/sortilin and mBDNF/TrkB signaling pathways is dysregulated^32^. In another meta-analyses study, serum levels of mature BDNF in depression patients are lower than normal people while no difference of proBDNF are discovered^33^. Though there are inconsistent serum results of proBDNF levels in depression patients, the values of proBDNF/mBDNF is found lower in depression patient^34^. The role of proNGF/mature NGF in etiology of depression disorder is not sufficient to be studied compared with that in AD^35^. However, in our studies, it was found that stress procedures might also inhibit the conversion of pro-NGF to NGF in hippocampus on animals, which was similar to BDNF on clinical studies. The insufficient supplies of neurotrophins cannot satisfy the demand of nourishment required by growth of healthy neuron while over expressions of pro-neurotrophins initiate cell death, which deteriorates the normal neurogenesis and then depression disorder or AD happens^36^. Therefore, KXS series formulae might exert anti-depressive efficacy by modulating metabolite pathway of neurotrophins, i.e. enabling transformation of pro-neurotrophins to its mature form and therefore restoring the decreased amounts of neurotrophins.

In addition to neurotrophin expression induction by KXS, Hu *et al*. also explored the role of BDNF in KXS exerting anti-depressive efficacy both *in vivo* and *in vitro* studies^37^. On a BDNF knock down primary hippocampal neuronal culture and rats mediated by lenti-viral vectors transfection and dentate gyrus injection, KXS can attenuate primary hippocampal neuronal cell death and exert anti-depressive effect in sucrose consumption tests and open field tests. In biochemical tests, BDNF reduction by LV-shBDNF RNA interference can be successfully reversed by KXS either *in vitro* or *in vivo*. These findings imply that KXS can un-regulate BDNF expressions and the composition and preparation of KXS by Hu *et al*. is same to D-652 in our studies. Therefore, our studies provided substantial evidences for KXS inducing neurotrophin expression and supported neurotrophin as an important target for anti-depressant interventions.

Among three ratios of KXS series formulae, KXS with higher amount of GR and PR seemed to exert better anti-depressive efficacy and more power in regulating neurotrophin metabolite pathway. These phenomena were also in consistent with our previous studies of KXS on inducing synthesis and secretion of neurotrophic factor, such as NGF, BDNF, GDNF and NT3, on rat astrocyte primary cultures^38^. The constituents of KXS beneficial for neurotrophin expressions has also been reported. In CUMS induced depressive rats, total saponins of GR can reverse decreased BDNF level induced by stress procedures^39^. In parallel, ginsenoside Rg_1_ up-regulates the BDNF signaling pathway in hippocampus of mice and Re, Rd induce TrkA gene expressions in Neuro-2a cells^39–41^. In addition, 3, 6′-disinapoyl sucrose derived from PR reverses the reduced BDNF levels in stress-induced depressive rats^42^, while saponins from PR enhance the production of NGF in rat astrocytes^43^. Eugenol derived from ATR increases BDNF mRNA expression level in hippocampus of mice^44^. Among the tested three KXS formulae in current studies, the amounts of GR and PR were the highest in D-652, which therefore might explain the best effect of this ratio in enhancing expressions of neurotrophins and Trk receptors. However, the effect of D-652 was only evaluated on stress-related depression animal model here. Therefore, it might unsuitable to conclude that the anti-depressive effect of D-652 was superior to other ratio by only one model. Indeed, different ratios of KXS formulae were all applied on clinic. In addition, it could not be ignored that KXS might depend on multiple compounds with synergistic or antagonistic mechanisms which modulated the complex signaling networks directly or indirectly, and the concrete mechanism and active compounds should be further explored.

Besides, though a previous publication reports a lower dose of KXS effectively shortens the immobile time in forced swimming test, this phenomenon could not be found in our test. The differences of animal species, drug dosages and experimental conditions might lead to this discrepancy^16^. In open field test, it seemed that KXS might increase mobility according to the increased travelled distances, which might influence the immobile swimming time in FST test. Combined with increased time in the chamber center, whether KXS might induce anxiolytic effect should also be explored. In addition, in the exploration of K252a and tPA-stop treatment on the antidepressant-like effect of D-652 via forced swimming test, whether the locomotor activity was altered along with the prolonged immobile time was not evaluated by locomotor activity measurements. Therefore, the relationship between neurotrophic factor regulation, locomotor activity and depressive behaviors should be explored in more details in future studies.

## Methods

### Materials

tPA-stop was purchased from American Diagnostica Inc. (Stamford, CT) and K252a was purchased from Sigma-Aldrich (St. Louis, MO). In addition, the chemicals and instruments not specially mentioned were purchased from Sigma-Aldrich (St. Louis, MO).

### Preparation of herbal extracts

The extracts of KXS series formulae were prepared according to the historical documents listed in Table 1. The following dried raw materials: Ginseng Radix et Rhizome (root and rhizome of *P*. *ginseng*), Polygalae Radix (root of *P*. *tenuifolia*), Acori Tatarinowii Rhizoma (rhizome of *A*. *tatarinowii*), and Poria (sclerotium of *P*. *cocos*), were all purchased from Tianling Company of Chinese herbs in Suzhou China, which were authenticated by associate Prof. Hui Yan of Nanjing University of Chinese Medicine, according to their morphological characteristics. The details of decoction preparation and quality control could be referred to the previous published work^45^. For quality control, a HPLC-DAD-MS/MS fingerprint of KXS was shown in Supplementary Fig. 1. By determining the amounts of marker chemicals from each herb, a standardized KXS extract should contain no less than the listed amounts of detected chemical markers in Supplementary Table. These parameters established the chemical standards of KXS for subsequent studies on biological studies.

**Table 1:** Historical record of KXS series formulae.

Notation	Record	Ratio			
		GR	PR	ATR	PO
K-652	*Beiji Qianjin Yaofang*	1	1	25	50
K-984	*Yixin Fang*	1	1	1	2
D-652	*Beiji Qianjin Yaofang*	3	2	2	3

### Animals and house conditions

Male ICR mice weighing 20–22 g were obtained from the Laboratory Animal Services Center, Nanjing University of Chinese Medicine. Animals were maintained on a 12 hours’ light/dark cycle (lights on at 6:00 a.m., lights off at 6:00 p.m.) under controlled temperature (22 ± 2 °C) and humidity (50 ± 10%), and were given standard diet and water ad libitum. They were allowed to acclimatize for 7 days before model development. The experiments on animals have been approved by the Animal Experimentation Ethics Committee of Nanjing University of Chinese Medicine and conformed to the guidelines of the “Principles of Laboratory Animal Care” (NIH publication No. 80-23, revised 1996). Effort was made to minimize the number and suffering of the animals.

### Chronic unpredictable mild stress procedure

The procedures of CUMS were performed as described^46^. In brief, the CUMS protocol consisted of the sequential application of a variety of mild stressors: (1) food deprivation for 24 hours, (2) water deprivation for 24 hours, (3) electric shock (30 V, 5 secs), five times, (4) shaking for 0.5 hours (160 Hz), (5) overnight illumination, (6) soiled cage (200 mL water in 100 g sawdust bedding) for 24 hours, (7) physically restraint for 2 hours. These stressors were randomly scheduled over a one-week period and repeated throughout the 4-week experiment. Non-stressed animals were left undisturbed in their home cages except during housekeeping procedures such as cage cleaning.

### Drug treatment

Mice were randomly divided into six groups of eight individuals. The control animals were given with saline. For another five groups, the animals were treated with CUMS procedures for 4 weeks. The details of CUMS were shown in Supplementary Fig. 2. Afterwards, these CUMS treated mice were given different drugs intragastrically for 7 days accompanied with CUMS procedures. The positive drug group of CUMS-treated mice were treated with fluoxetine (4 mg/kg), a well-known selective serotonin reuptake inhibitor antidepressant purchased from Sigma-Aldrich (St. Louis, MO). Another three groups were treated with different KXS extracts at 1.5 g/kg/day (converted to crude herbal materials at 10 g/kg/day). For the CUMS group, the mice were only supplied with saline.

To explore the role of neurotrophic factor regulation in anti-depressive effect of KXS, CUMS depressive mice were treated with corresponding blockers in addition to KXS treatment. After 4-week CUMS exposure, blockers were treated together with KXS extracts for 7 days. As to neurotrophic factor synthesis, tPA-stop (1.5 μg/kg, dissolved in 5 μL artificial CSF) was transcranial bilateral injected in hippocampus (anterior-posterior (AP) = −2.3 mm, medial-lateral (ML) =  ±1.8 mm from the bregma and dorsal-ventral (DV) = 2.0 mm from cerebral dura mater, which were standardized from the stereotaxic atlas of Paxinos and Watson) for three times (every other day, 2.5 μL per-side). The location of intra-hippocampal injections was verified in each animal used in the experiment. For Trk receptors, K252a, was administered intraperitoneally (i.p.) at 25 μg/kg every day. Behavioral and biochemical tests were carried out after treatment of KXS and blockers. Controlled animals were given the corresponding vehicle, also in the same volume.

### Behavioral tests

Sucrose preference test was carried out at the end of 4 weeks CUMS exposure. Briefly, 72 hours before the test, mice were trained to adapt to 1% sucrose solution (w/v): two bottles of 1% sucrose solution were placed in each cage. After 24 hours, 1% sucrose in one bottle was replaced with tap water. After the adaptation for 24 hours, mice were deprived of water and food for another 24 hours. Sucrose preference test was conducted at 9:00 a.m., in which mice were housed in individual cages and were free to access to two bottles containing either 100 ml of sucrose solution (1%, w/v) or 100 ml of water, respectively. After 3 hours, the weights of consumed sucrose solution and tap water were recorded and the sucrose preference was calculated by the following formula: sucrose preference = sucrose consumption/(tap water consumption + sucrose consumption) × 100%.

Forced swimming test was carried out after the 24 hours of sucrose preference test. The test was performed according to the previous reported method^47^. Briefly, mice were forced to swim in a transparent glass vessel (20 cm high, 14 cm in diameter, filled with 10 cm of water at 24–26 °C) placed in a cabinet. The motilities of mice were recorded with a camera and mice were considered immobile when they made no attempts to escape except the movements necessary to keep their heads above the water. The total duration of immobile time (seconds) was recorded during the last 4 min of a single 6-min test session while initial 2 min was applied for mice adaption.

Locomotor activity was evaluated by open-field test, which was performed as described by Xue *et al*. and modified^48^. In brief, the test was carried out in a well-illuminated (~300 lux) transparent acrylic cage (40 × 40 × 15 cm). The activities of mice in two compartments, a compartment near the walls and a central area compartment, were tracked. The time spent in central compartment, the distance travelled (cm) and the number of rearing were analyzed. To avoid the possible disturbance, testing apparatus was thoroughly cleaned with 70% ethanol and dried after each occupancy. The test was carried out at three time points: before CUMS exposure, at the end of 28-day CUMS exposure and after drug treatment.

### ELISA assays

After forced swimming test, the mice were sacrificed by rapid decapitation, and the hippocampus were rapidly removed and frozen in liquid nitrogen and kept in −80 °C until assay. For the lysate preparation, the tissues were homogenized in lysis buffer (10 mM HEPES, pH 7.5, 1 M NaCl, 1 mM EDTA, 1 mM EGTA, 0.5% Triton X-100, 5 mM benzamidine HCl, 10 μM aprotinin, 10 μM leupeptin). Lysates were centrifuged 15,000 × *g* for 30 minutes at 4  °C. The supernatant was separated and stored at −20 °C until the assays. Protein levels of NGF and BDNF were measured using commercially available ELISA kits (Mouse NGF LF-EK 50290, Mouse BDNF LF-EK 50013, Abfrontier, Korea) according to the manufacturer’s instructions. All samples were measured in duplicate in the same assay to minimize inter-assay variation. The ranges of the calibration curve were 7.8–500 pg/ml. The protein content was expressed as ng/g wet weight of tissue.

### Western blot analysis

The details of SDS-PAGE and western blot analysis could be referred to the previous published work^49^. The total protein samples were loaded on the 15% polyacrylamide gels and separated. The primary antibodies used were: rabbit polyclonal anti-NGF (H-20, 1:1000, Santa Cruz Biotechnology), rabbit polyclonal anti-BDNF (N-20, 1:1000, Santa Cruz Biotechnology), rabbit polyclonal anti-TrkA (2505, 1:1000, Cell Signaling Technology), rabbit polyclonal anti-TrkB (4603, 1:1000, Cell Signaling Technology), mouse monoclonal anti-α tubulin (T-6074, 1:10000, Sigma-Aldrich) at 4 °C temperature overnight. The bands were compared on an image analyzer, using in each case a calibration plot constructed from a parallel gel with serial dilution of one of those samples: this was to ensure the sub-saturation of the gel exposure. Gel documentation and relative quantification were performed with Image-J Digital Imaging System.

### Real-time quantitative PCR

To quantify the mRNA expression levels of the regulation genes of tyrosine receptor kinases and neurotrophic factor metabolism, total RNA from brain tissues were isolated by Trizol reagent (Invitrogen, Carlsbad, CA) according to the manufacture’s instruction. Up to 1 μg of total RNAs isolated from tissues were reverse transcribed by using PrimeScript^TM^ RT reagent Kit with gDNA Eraser (Takara, Japan) according to the manufacture’s instruction, and the cDNA of each sample was obtained. Real-time quantitative PCR was performed on equal amounts of cDNA by using SYBR Green Master mix with Rox reference dye (Roche, Germany). The SYBR green signal was detected by Applied Biosystems 7500 fast real-time PCR system. Transcript levels were quantified by using the ΔΔCt value method^50^. PCR products were analyzed by gel electrophoresis, and the specificity of amplification was confirmed by the melting curves. The primers were as follows: 5′-GAC TCA AGG GAC TTT CGG TG-3′ (sense primer, S) and 5′-CTC GAA GCA AAC CAG AGG TC-3′ (anti-sense primer, AS) for Plasminogen (188 bp; NM_008877.3); 5′-TGA CAA CGA CAT CGC ATT AC′-3′ (S) and 5′-TTC AGC CGG TCA GAG AAG-3′ (AS) for tPA (186 bp; NM_008872.3); 5′-TTG GCC CTC ATC AAT GCT GTA′-3′ (S) and 5′-AGA TAC CAC CAG CCT CAT TGG-3′ (AS) for neuroserpin (184 bp; AJ001700.1); 5′-CTT TGA GGA TCC GCA GAC C′-3′ (S) and 5′-CTG ACG TGG GTT ACC TCT G-3′ (AS) for MMP9 (132 bp; BC046991.1); 5′-CTT TGA GGA TCC GCA GAC C′-3′ (S) and 5′-AGC TGT TGC TGA CAA GAT GGT GGT-3′ (AS) for TIMP-1 (180 bp; U54984.1); 5′-AAC GGA TTT GGC CGT ATT GG-3′ (S) and 5′-CTT CCC GTT CAG CTC TGG G-3′ (AS) for GAPDH (195 bp; AF106860.2).

### Data analysis

Multiple comparisons were made by using one-way or two-way ANOVA followed by a Bonferroni post hoc analysis if appropriate (version 13.0, SPSS, IBM Corp., Armonk, NY, USA). Before ANOVA analysis, normal distribution test was carried out firstly. The control group was varied in different experiments, and which was specified in the figure legends. Data were expressed as Mean ± SEM, where n = 8. Statistically significant changes were classed as significant [*] where p < 0.05, highly significant [**] where p < 0.01.

## Electronic supplementary material

Supplementary information
