# Supplementary material for: Kai-Xin-San series formulae alleviate depressive-like behaviors on chronic mild stressed mice via regulating neurotrophic factor system on hippocampus
Source: Sci Rep. 2017 May 3;7:1467. doi: 10.1038/s41598-017-01561-2 (PMC5431115; doi:10.1038/s41598-017-01561-2)
Supplement: Supplementary file 1 — Supplementary information [file 41598_2017_1561_MOESM1_ESM.doc]

**Kai-Xin-San series formulae alleviate depressive-like behaviors on chronic mild stressed mice via regulating neurotrophic factor system on hippocampus.**

Yue ZHU* ^a^, Cheng CHAO ^a^, Xiuzhu DUAN ^a^*,* Xiaoxuan CHENG ^a^, Pei LIU ^a^, Shulan SU ^a^, Jinao DUAN* ^a^, Tina Tingxia DONG^b^, Karl Wah-Keung TSIM^b^

**Supplementary Figure 1: Chemical fingerprint chromatograms of KXS formulae.**

**(A):** Fingerprint chromatograms of KXS formulae were made by HPLC-DAD at wavelength of 330 nm. The identification of 3, 6’-disinapoyl sucrose (1), α-asarone (8) and β-asarone (7) were shown in the chromatogram. **(B):** Fingerprint chromatograms of KXS were made by HPLC-MS/MS method at negative scan mode. The identification of ginsenoside Rg_1_ (2), Re (3), Rb_1_ (4), Rd (6) and pachymic acid (9) were shown in the chromatogram. The internal marker control was astragaloside IV (5). Representative chromatograms are shown, *n* = 3.

**Supplementary Figure 2: Schedule of chronic mild stress (CMS) procedure.**

The CMS protocol consisted of the sequential application of a variety of mild stressors. These stressors were randomly scheduled over a one-week period from Day 1 to Day 7 and repeated for 4-week during the entire experiment.

**Supplementary Table 1 Criteria for standardized KXS series formulae**

| **Marker chemical** | **K-652** | **K-984** | **D-652** |
| --- | --- | --- | --- |
| Ginsenoside Rb_1_ | 20.4 ± 1.7 ^a^ | 184.1 ± 8.0 | 190.4 ± 2.6 |
| Ginsenoside Rd | 8.0 ± 0.6 | 66.7 ± 4.5 | 68.4 ± 5.8 |
| Ginsenoside Re | 19.0 ± 1.3 | 165.5 ± 6.8 | 154.0 ± 8.4 |
| Ginsenoside Rg_1_ | 24.6 ± 2.2 | 196.6 ± 7.0 | 181.2 ± 5.9 |
| 3, 6’-disinapoyl sucrose | 33.6 ± 0.3 | 283.8 ± 0.9 | 179.9 ± 1.8 |
| α-Asarone | 51.4 ± 0.2 | 30.4 ± 5.9 | 28.9 ± 3.3 |
| β-Asarone | 1112.4 ± 1.9 | 489.5 ± 7.3 | 499.1 ± 2.8 |
| Pachymic acid | 21.1 e^-3^ ± 0.4 e^-3^ | 17.7 e^-3^ ± 0.7 e^-3^ | 8.1 e^-3^ ± 0.8 e^-3^ |

^a^ Values were expressed in mg/100g of dried powder of KXS, Mean ± SEM, n=3.
